# Evaluation of tricuspid valve regurgitation following transvenous rotational mechanical lead extraction

**DOI:** 10.1093/europace/euae191

**Published:** 2024-07-11

**Authors:** Federico Migliore, Raimondo Pittorru, Manuel De Lazzari, Pietro Bernardo Dall’Aglio, Antonella Cecchetto, Marco Previtero, Valeria Pergola, Gaetano Thiene, Giulia Masiero, Giuseppe Tarantini, Vincenzo Tarzia, Gino Gerosa

**Affiliations:** Department of Cardiac, Thoracic, Vascular Sciences and Public Health, University of Padova, Via Giustiniani 2, 35128 Padova, Italy; Department of Cardiac, Thoracic, Vascular Sciences and Public Health, University of Padova, Via Giustiniani 2, 35128 Padova, Italy; Department of Cardiac, Thoracic, Vascular Sciences and Public Health, University of Padova, Via Giustiniani 2, 35128 Padova, Italy; Department of Cardiology and Medical Intensive Care, St. Josef Hospital Freiburg, Freiburg, Germany; Department of Cardiac, Thoracic, Vascular Sciences and Public Health, University of Padova, Via Giustiniani 2, 35128 Padova, Italy; Department of Cardiac, Thoracic, Vascular Sciences and Public Health, University of Padova, Via Giustiniani 2, 35128 Padova, Italy; Department of Cardiac, Thoracic, Vascular Sciences and Public Health, University of Padova, Via Giustiniani 2, 35128 Padova, Italy; Department of Cardiac, Thoracic, Vascular Sciences and Public Health, University of Padova, Via Giustiniani 2, 35128 Padova, Italy; Department of Cardiac, Thoracic, Vascular Sciences and Public Health, University of Padova, Via Giustiniani 2, 35128 Padova, Italy; Department of Cardiac, Thoracic, Vascular Sciences and Public Health, University of Padova, Via Giustiniani 2, 35128 Padova, Italy; Department of Cardiac, Thoracic, Vascular Sciences and Public Health, University of Padova, Via Giustiniani 2, 35128 Padova, Italy; Department of Cardiac, Thoracic, Vascular Sciences and Public Health, University of Padova, Via Giustiniani 2, 35128 Padova, Italy

**Keywords:** Complications, Mechanical lead extraction, Transvenous lead extraction, Tricuspid valve regurgitation, Tricuspid valve damage

## Abstract

**Aims:**

Transvenous lead extraction (TLE) is potentially complicated by significant tricuspid valve regurgitation increase (TRI). However, there are limited data on the effect of the bidirectional rotational mechanical sheaths on significant TRI. The aim of the present study was to investigate the rate of significant changes in tricuspid regurgitation (TR) severity following mechanical rotational TLE and their outcomes.

**Methods and results:**

In 158 patients (mean age 66 ± 16.9 years) undergoing mechanical rotational TLE, acute changes in TR severity were assessed by echocardiography evaluation. A significant acute TRI was defined as an increase of at least one grade with a post-extraction severity at least moderate. A total of 290 leads were extracted (mean implant duration, 93 ± 65 months). Significant TRI was noted in 5.7% of patients, and it was linked to tricuspid valve damage, TLE infection indication, and longer lead implant duration. Univariate predictors of significant TRI included implant duration of all leads [odds ratio (OR) 1.01; 95% confidence interval (CI) 1.003–1.018; *P* = 0.001] and right ventricular leads (OR 1.01; 95% CI 1.004–1.017; *P* = 0.002). Severe increase of TR following TLE was an independent predictor of mortality [hazard ratio (HR) 5.20; 95% CI 1.44–18.73; *P* = 0.012 ] along with severe systolic dysfunction (HR 2.37; 95% CI 1.01–5.20; *P* = 0.032), and systemic infection (HR 2.28; 95% CI 1.06–4.89; *P* = 0.035).

**Conclusion:**

Significant TRI was detected in 5.7% of patients following transvenous rotational mechanical lead extraction. The duration of lead implantation emerged as the sole predictor of significant TRI. Physicians engaged in TLE should exercise greater vigilance for this potential complication.

## Graphical Abstract

**Graphical Abstract:**
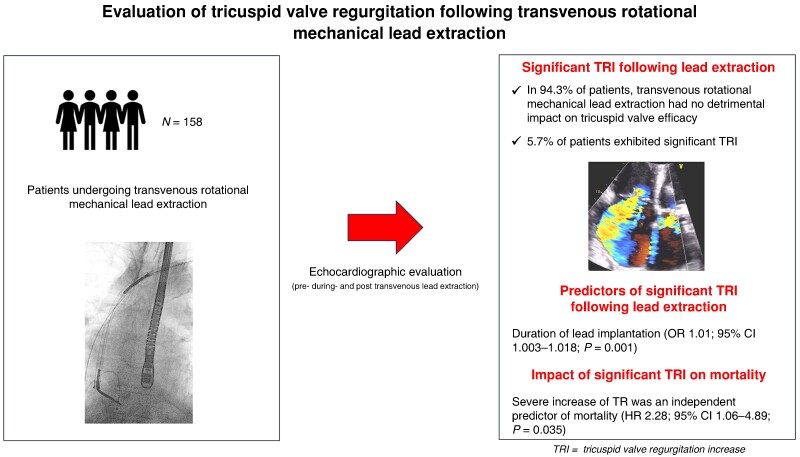

**What’s new?**

- In 94.3% of patients, transvenous rotational mechanical lead extraction had no detrimental impact on tricuspid valve (TV) efficacy; however, 5.7% of patients exhibited significant tricuspid regurgitation increase.
- Non-urgent TV replacement was carried out in 1.9% of patients, primarily due to TV damage.
- The duration of lead implantation emerged as the only predictor of significant tricuspid regurgitation increase following transvenous rotational mechanical lead extraction.
- Severe increase of tricuspid regurgitation after lead extraction was an independent predictor of mortality.
- Physicians engaged in transvenous lead extraction should exercise greater vigilance for this potential complication.

## Introduction

Transvenous lead extraction (TLE) is an integral part of the lead management strategy, and it has been established to be a safe and effective method of lead removal with a high rate of procedural success and low rate of major complications regardless of the technique and tools used.^1–3^ Injury to the superior vena cava (SVC) is the most dangerous and feared complication of TLE.^1–3^ However, TLE procedure is potentially complicated by significant tricuspid valve (TV) injury with a subsequent increase in tricuspid regurgitation (TR).^4–9^ Recent data reported that significant TR and TV damage after TLE are an increasingly recognized complication of TLE, especially if the procedures are monitored by transoesophageal echocardiography (TEE).^10,11^ Therefore, non-routine use of cardiac imaging before, during, and after the procedure may be a possible cause of underdiagnoses of TV regurgitation in patients undergoing TLE. The most significant factors that may increase the risk of significant acute TR increase are the presence of significant fibrotic encapsulation of the right ventricular (RV) leads, complex extraction procedures, especially when using laser sheaths and longer implant duration.^4,10,11^ However, there are limited data on the effect of the bidirectional rotational mechanical sheaths on TR severity. The aim of this single-centre study was to investigate the rate of significant changes in TR severity following mechanical rotational TLE and their outcomes.

## Methods

### Study population

The study population consisted of patients undergoing transvenous rotational mechanical lead extraction by exclusively using the Evolution RL rotational sheaths and different mechanical ancillary tools (Cook Medical, Bloomington, IN, USA), supporting the procedure from March 2015 to December 2023. We did not employ any other lead extraction tool, including laser technique. We included only patients who underwent extraction of ≥1 RV pacing or defibrillator lead. Patients (i) with recently implanted leads (implant duration < 12 months); (ii) whose leads had been explanted by simple traction without or with locking stylet or Bulldog Lead Extender (Cook Medical); (iii) who underwent extraction of atrial leads alone; and (iv) who underwent extraction of proximal remnant of RV lead dissected above the TV were excluded. Furthermore, patients with incomplete echocardiographic examinations were excluded from the analysis. The study was conducted in compliance with the principles outlined in the Declaration of Helsinki and approved by the local medical ethics committee. The device manufacturer did not sponsor or influence the study in any way.

### Extraction procedure

All procedures were performed by two electrophysiologists experienced in TLE in the operating room, hybrid room, or electrophysiology laboratory under general anaesthesia or sedation with continuous electrocardiographic monitoring of arterial blood pressure. Standby cardiac surgery was always available. Cardiac surgeon was a designated operator familiar with the management of lead extraction procedure and complications. In patients dependent on bradycardia support, a temporary pacemaker (PM) was inserted through the femoral vein. If present, the active fixation mechanism was retracted and manual traction was attempted. If this was unsuccessful, manual traction was attempted again using a locking stylet (Liberator, Cook Medical). When manual traction was ineffective, fibrous adhesions surrounding the lead were dissected using the Evolution RL rotational sheaths with available extraction tools (Evolution Shortie RL, One-Tie Compression Coil, and SteadySheath Evolution tissue stabilization sheath; Cook Medical) with a stepwise approach as previously reported in detail^12^ in all procedures. Free-floating leads and remnants after extraction were snared via a right jugular or femoral approach depending on how the leads were positioned using a Needle’s Eye Snare (Cook Medical). All patients were monitored for procedure-related complications at the time of extraction, during their hospital stay. Success and failure were defined according to the definitions of the 2017 Heart Rhythm Society and the 2018 European Heart Rhythm Association expert consensus.^1,2^ Complete procedural success rate, clinical success rate, and lead removal with clinical success were reported. Major complications were defined as outcomes that were life-threatening, resulted in significant or permanent disability or death, or required surgical intervention, and minor complications were defined as events related to the procedure that required medical intervention or minor procedural intervention.^1,2^

### Echocardiographic evaluation

According to the protocol of our department, routine transthoracic echocardiography (TTE) is mandatory before TLE and after the procedure before discharge in all patients for the evaluation of underlying cardiac disease, including RV and left ventricular systolic function and abnormalities of the valves, with particular attention to the TV function. Additionally, for this analysis, intraprocedural TEE was also performed in all cases. Transthoracic echocardiography allowed to evaluate the pre-operative status of pericardial effusion, assess the structural integrity and function of the cardiac valves apparatus including the TV, describe vegetations on the valves or leads when endocarditis was suspected, and visualize direct pulling on the heart and the RV cavity during lead removal. Pre–TLE and post–TLE TR severities were graded as trivial, mild, moderate, or severe according to international guidelines.^13,14^ A significant acute tricuspid regurgitation increase (TRI) was defined as an increase of at least one grade with a post-extraction severity at least moderate. The degree of TR was performed on the basis of a multiparametric evaluation and not on the basis of a single echocardiographic evaluation by two experienced operators. Damages of the leaflets and subvalvular apparatus were also investigated. According to the definitions of the 2017 Heart Rhythm Society and the 2018 European Heart Rhythm Association expert consensus, worsening of TR was considered a minor complication and TV damage requiring intervention was considered as a major complication. For the present study, we used Philips IE33 and Philips EPIQ System Philips Medical System, MA, USA, for TEE and GE vivid E9, GE Vingmed Ultrasound, Horten, Norway, and Philips EPIQ System Philips Medical System for TTE.

### Statistical analysis

Continuous variables are expressed as mean ± standard deviation (SD) or median values and 25th and 75th percentiles. Categorical variables are presented as actual numbers and frequencies. Shapiro–Wilk *W* test was used to assess the normality of continuous variables. Categorical differences between groups were evaluated by using the *χ*^2^ test or the Fisher’s exact test as appropriate. Comparison of two groups of categorical variables were performed using Student’s *t*-test of unpaired samples, and in case of non-normality or small sample size, Mann–Whitney *U* test was used. Univariate analysis was performed using logistic regression with the clinical and device- or procedure-related variables to assess the risk factors for the significant TRI and to calculate adjusted odds ratios (ORs) and their 95% confidence intervals (CIs). Patients were followed up with serial outpatient evaluations or with telephone interviews to determine whether they experienced any adverse events. Univariate and multivariate analyses were performed to identify the predictors of mortality. A two-tailed *P* < 0.05 was considered statistically significant. All analyses were performed using SPSS statistical software package (version 21.0; SPSS Inc., Chicago, IL, USA).

## Results

### Study population

The study population comprised 158 patients [79% male; mean age 66 ± 16.9 years, median 67 (57–79)]. Patient and lead characteristics are reported in *Table 1*. Indications for TLE included infection in 100 cases (63%), lead malfunction in 52 (33%), system upgrade in 3 (2%), and lead-related TR in 3 (2%). Patients with CIED infection were treated according to current guidelines and infectious disease consultation.

**Table 1:** Baseline clinical characteristics of patients with and without significant tricuspid regurgitation increase

	Overall (*n* = 158)	No significant	Significant	*P*-value
		TR increase (*n* = 149)	TR increase (*n* = 9)	
Age, years	67 (57–79)	67 (57–79)	74 (70–86)	0.08
Male	125 (79)	118 (79)	7 (77)	1
Body mass index, kg/m^2^	25 (22–29)	25 (22–29)	23 (21–27)	0.18
Hypertension	99 (62)	92 (61)	7 (77)	0.48
Diabetes	50 (31)	47 (31)	3 (33)	1
Chronic kidney disease	22 (13)	19 (12)	3 (33)	0.11
Ischaemic cardiomyopathy	32 (20)	31 (20)	1 (11)	0.68
Non-ischaemic cardiomyopathy	38 (24)	34 (22)	4 (44)	0.22
Previous open-heart operation	32 (20)	30 (20)	2 (22)	1
Previous valve surgery	15 (9)	15 (10)	0	0.48
Congenital heart disease	7 (4)	7 (4)	0	1
Echocardiographic findings				
LVEF, %	50 (37–58)	50 (37–58)	55 (37–64)	0.39
EF ≤ 35%	32 (20)	30 (20)	2 (22)	1
FAC	42 (36–47)	42 (36–47)	42 (37–49)	0.55
RV end-diastolic area (cm^2^/m^2^)	10 (7–13)	10 (7–13)	12 (10–14)	0.121
Indication for lead extraction				
Infection	100 (63)	91 (61)	9 (100)	0.02
Lead malfunction	52 (33)	52 (34)	0 (−)	0.03
Other causes^a^	6 (3)	6 (4)	0 (−)	1

Values are median (interquartile range) or *n* (%). LVEF, left ventricular ejection fraction; RV, right ventricular; RVFAC, right ventricular fractional area change; TR, tricuspid regurgitation.

^a^System upgrade, *n* = 3, lead-related tricuspid regurgitation, *n* = 3.

### Procedural data

A total of 290 leads were treated [mean number 1.9 ± 0.9 per patient, median 2 (2–3) range 1–5]. Of the target extracted leads, 96 (33.2%) were right atrial leads, 70 (24.1%) were RV pacing leads, 29 (10%) were coronary sinus leads for cardiac resynchronization therapy, 6 (2%) were VDD leads, 6 (2%) were conduction system pacing leads, and 83 (28.7%) were implantable cardioverter-defibrillator (ICD) leads (35 dual-coil, 48 single-coil). The leads with a passive fixation were 168 (57.9%). The mean implant duration was 93 ± 65 months [median 79 (42–135) months]. Occlusion of the left subclavian vein was observed in 21 patients (13.2%). The additional use of snares (Needle’s Eye Snare, Cook Vascular) via the femoral approach was required in 7 patients (4.4%). On average, 3 ± 1 tools [median 3 (2–4), range 2–6] including locking stylet, One-Tie Compression Coil, Bulldog Lead Extender, Evolution Shortie RL, Evolution RL, and SteadySheath Evolution tissue stabilization sheath were used. Two hundred and seventy-five (94.8%) were completely extracted, whereas incomplete removal was observed in 15 leads (5.2%) among 15 patients (9.4%). Of note, among 15 leads incompletely removed, we observed retention of <1 cm remnant of lead material consisting of a small tip of the lead or the screw in 9 patients. The lead fragments did not result in any undesired outcomes or lead extraction-related complications. Complete failure of lead extraction was seen in 5 (1.7%) leads among 4 patients (2.5%). Complete procedural success rate, clinical success rate, and lead removal with clinical success rate were 90.5% (143/158), 97.5% (154/158), and 98.3% (285/290), respectively. Major complications related to the TLE procedure occurred in 5 patients (3%): haemopericardium requiring prompt surgical drainage (*n* = 2) and TV damage requiring intervention (*n* = 3). No injury to the SVC occurred in any cases and no procedure-related deaths were reported. Twenty-two minor complications (13.9%) were reported, including significant worsening TV function (*n* = 6), haematoma at the pocket requiring drainage (*n* = 6), pericardial effusion without need for evacuation (*n* = 3), femoral vascular lesion with need for intervention (*n* = 1), arterovenous fistulae with need for treatment (*n* = 2), bleeding with need for transfusion (*n* = 2), and venous thrombosis requiring treatment (*n* = 2).

### Significant tricuspid regurgitation increase following transvenous lead extraction

**Figure 1:**
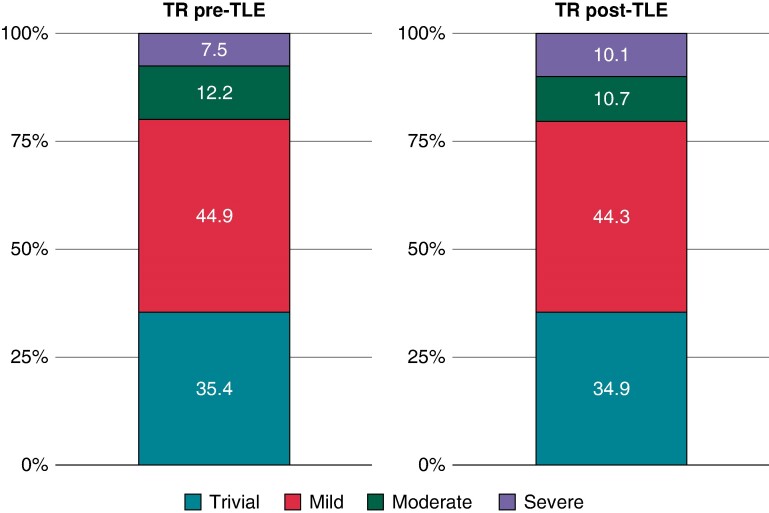
Grading of TR before and after transvenous rotational mechanical lead extraction. TR, tricuspid regurgitation; TLE, transvenous lead extraction.

**Figure 2:**
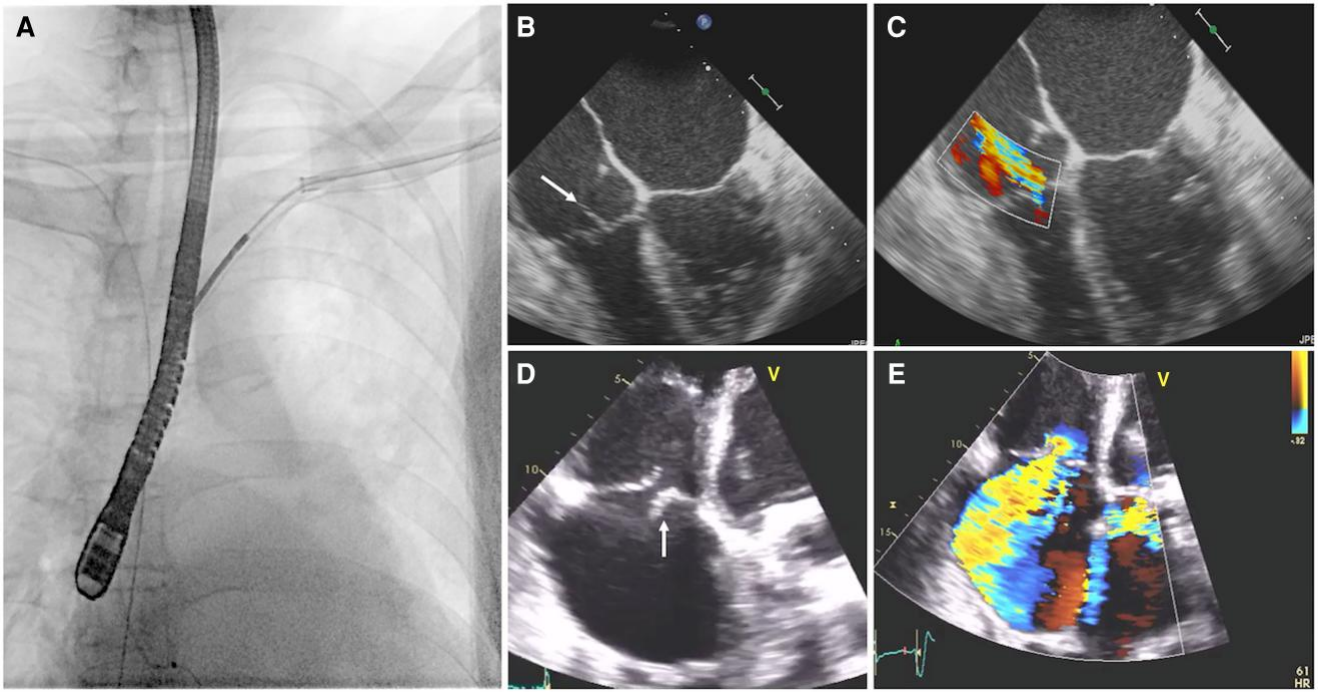
Echocardiography evaluation following TLE showing severe TR and flail tricuspid leaflet (white arrow). (*A*) Transvenous rotational mechanical lead extraction. (*B* and *C*) TEE evaluation immediately after the procedure. (*D* and *E*) TTE evaluation after the procedure confirming severe TR and flail tricuspid leaflet. TEE, transoesophageal echocardiography; TTE, transthoracic echocardiography.

Significant TRI was observed in 9 patients (5.7%) after TLE (severe degree, *n* = 5; moderate degree, *n* = 4). Tricuspid valve damage was observed in 3 patients. The details of change in TR severity detected after TLE are shown in *Figure 1* and Supplementary material online, *Figure S1*. Baseline and procedure characteristics of the overall study population and of patients with and without significant TRI are reported in *Tables 1* and *2*. Patients who experienced significant TRI were more likely to have an infection indication (*P* = 0.02) and have a significant longer median implant duration both of all leads and RV leads compared to patients without significant TRI (*P* = 0.003 and *P* = 0.001 respectively). There was a trend towards the use of the SteadySheath Evolution tissue stabilization sheath (*P* = 0.06). There were no other significant differences in baseline clinical and technical characteristics between patients with and without significant TRI. Evidence of TV damage was found more frequently in the significant TRI group (*P* = 0.005; see *Figure 2*). Clinical and technical characteristics and outcome of patients who reported significant TRI following TLE are reported in the Supplementary material online, *Table S1*. Among overall population, the mean procedural length was 155 ± 88 min. No statistically significance difference was observed between patient with and without TRI following TLE (161 ± 95 min vs. 154 ± 88 min; *P* = 0.90). Among the 9 patients who developed significant TRI, 4 received surgical repair indication: severe TR from TV damage (*n* = 3; Patient 4 was not a candidate for cardiac surgery due to significant comorbidities) and severe TR without TV damage (*n* = 1; see Supplementary material online, *Table S1* for more details). Overall, non-urgent TV replacement after multidisciplinary evaluation was performed in 3 patients (1.9%) with severe TR after TLE. The remaining 5 patients were medically managed with diuretic initiation or dose adjustments.

**Table 2:** Extracted leads and procedure characteristic of patients with and without significant tricuspid regurgitation increase

	Overall (*n* = 158)	No significant	Significant	*P*-value
		TR increase (*n* = 149)	TR increase (*n* = 9)	
PM	69 (43)	65 (43)	4 (44)	1
CRT PM	7 (4)	7 (4)	0	1
ICD	48 (29)	46 (31)	2 (22)	1
CRT defibrillator	35 (22)	32 (21)	3 (33)	0.41
Pacing device	76 (48)	72 (48)	4 (44)	1
ICD device	83 (52)	78 (52)	5 (55)	1
Implant duration of all extracted leads, months	79 (42–135)	76 (48–133)	143 (70–226)	0.003
Implant duration of RV leads, months	84 (44–138)	81 (42–134)	147 (86–214)	0.001
RV pacing lead alone	77 (49)	72 (48)	5 (55)	0.673
Defibrillator lead alone	76 (48)	72 (48)	4 (44)	1
RV pacing and defibrillator leads	5 (3)	5 (3)	0	1
Number of extracted leads (per procedure)	2 (1–2)	2 (1–2)	2 (1–3)	0.60
RV pacing passive lead ≥ 1 extraction	42 (26)	39 (26)	3 (33)	0.721
Passive fixation lead ≥ 1	106 (67)	100 (67)	6 (6)	1
Procedure with abandoned leads	16 (10)	14 (9)	2 (22)	0.22
Extraction tools				
Number of tools^a^	3 (2–4)	3 (2–4)	4 (2–5)	0.13
≥ 2 extraction tools^a^	96 (60)	90 (60)	6 (66)	1
SteadySheath Evolution tissue stabilization sheath	43 (27)	38 (25)	5 (55)	0.06
Use of snare	7 (4)	6 (4)	1 (11)	0.35
Extraction outcomes				
Complete procedural success	143 (90)	135 (90)	8 (88)	1
Lead removal with clinical success rate	285 (98)	268 (98)	17 (100)	1
Clinical procedural success rate	154 (97)	145 (97)	9 (100)	1
Use of AngioVac system	15 (9)	13 (8)	2 (22)	0.20
Echocardiographic findings				
Vegetation on TV or RV leads	30 (18)	27 (18)	3 (33)	0.37
TV damage	3 (2)	0	3 (33)	0.005
Procedural length, min	155 ± 88	154 ± 88	161 ± 95	0.90

Values are median (interquartile range) or *n* (%).

^a^At least one of the following tools: locking stylet, Bulldog Lead Extender, Evolution Shortie RL, Evolution RL, One-Tie Compression Coil or SteadySheath Evolution tissue stabilization sheath. CRT, cardiac resynchronization therapy; ICD, implantable cardioverter defibrillator; PM, pacemaker; RV, right ventricular; TR, tricuspid regurgitation; TV, tricuspid valve.

### Predictors of significant tricuspid regurgitation increase

Univariate predictors of significant TRI included implant duration of all leads (OR 1.01; 95% CI 1.003–1.018; *P* = 0.001) and RV leads (OR 1.01; 95% CI 1.004–1.017; *P* = 0.002).

### Transvenous lead extraction in patients with pre-existing severe tricuspid regurgitation

Twelve patients (7.5%) exhibited severe TR before TLE. However, among these, only 3 patients (1.8%) had a pre-existing severe lead-related TR requiring TLE for potential improvement in TV function. Post-extraction, 1 patient demonstrated a reduction in TR to a moderate level, while the remaining 2 patients showed no improvement in TR, and no iatrogenic damage to the TV was observed. One patient underwent edge-to-edge transcatheter repair (TriClip), and the other 2 opted for surgical repair of TR. Notably, 3 cases presented with significant tricuspid annular dilatation. In contrast, the remaining 9 patients without lead-related TR maintained severe regurgitation even after TLE.

### Mortality during follow-up

There were 2 in-hospital deaths (1.2%), one due to progressive heart failure (HF) and systemic infection and the other for COVID-19 pulmonary infection after TV replacement. No procedure-related mortality occurred. During a mean time follow-up of 30 ± 23 months, 29 patients (18.3%) died, for progressive congestive HF (*n* = 10), for progressive systemic infection (*n* = 8), for pulmonary infection due to COVID-19 (*n* = 2), and for non-cardiac causes (*n* = 9). Univariate predictors of mortality included ischaemic cardiomyopathy (*P* = 0.004), left ventricular ejection fraction ≤ 35% (*P* = 0.001), systemic infection (*P* = 0.002), and severe increase of TR after TLE (*P* = 0.01). At multivariable analysis, left ventricular ejection fraction ≤ 35% (*P* = 0.032), systemic infection (*P* = 0.035), and severe increase of TR after TLE (*P* = 0.012) remained independent predictors of mortality (see *Table 3*).

**Table 3:** Predictors of mortality

	Univariate analysis		Multivariate analysis	
	HR (95% CI)	*P*-value	HR (95% CI)	*P*-value
Ischaemic cardiomyopathy	2.93 (1.42–6.06)	0.004	–	–
LVEF ≤ 35%	3.28 (1.61–6.71)	0.001	2.37 (1.01–5.20)	0.032
TLE for systemic infection	3.09 (1.51–6.31)	0.002	2.28 (1.06–4.89)	0.035
Severe TR increase after TLE	4.84 (1.41–16.12)	0.01	5.20 (1.44–18.73)	0.012
Significant TRI after TLE	2.75 (0.96–7.87)	0.059	–	–

LVEF, left ventricular ejection fraction; TLE, transvenous lead extraction; TR, tricuspid regurgitation; TRI, tricuspid regurgitation increase.

## Discussion

The main findings of our study are as follows:

- In 94.3% of patients, transvenous rotational mechanical lead extraction had no detrimental impact on TV efficacy; however, 5.7% of patients exhibited significant TRI.
- Non-urgent TV replacement was carried out in 1.9% of patients with severe TR following TLE, primarily due to TV damage.
- The duration of lead implantation emerged as the only predictor of significant TRI following lead extraction.
- Severe increase of TR after TLE was an independent predictor of mortality along with severe systolic dysfunction and systemic infection.

### Tricuspid valve anatomy and physiology

**Figure 3:**
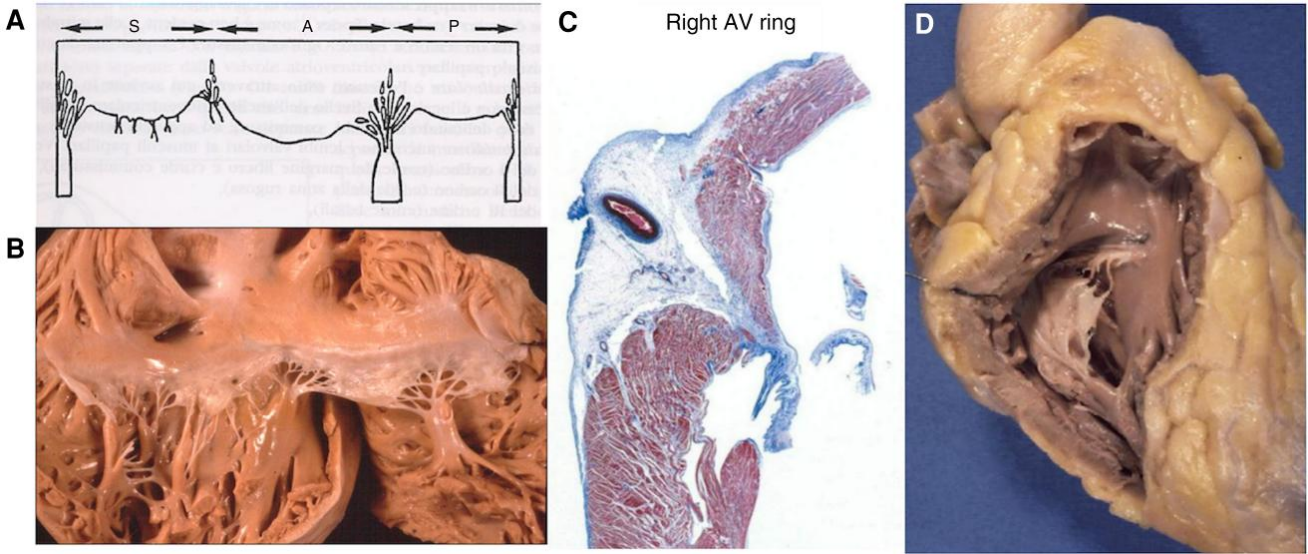
(*A* and *B*) The tricuspid valve: note the fan-like chordae at the anteroseptal and antero-postero-inferior commissures, arising from the tip of the Lancisi papillary muscle and the anterior papillary muscle, respectively. Look at the inferior papillary muscle with multiple pillars. Note the septal leaflet attached to the ventricular septum by chordae thendinae and tiny papillary muscles. (*C*) The fibrous annulus, to which a tricuspid leaflet inserts together with RV and right atrial myocardium. (*D*) Right ventricular outflow: note the trabecola septo-marginalis, the conal papillary muscle of Lancisi, the moderation band from the trabecula septo-marginalis, and the anterior papillary muscle. AV, atrio-ventricular.

The atrioventricular valve, in between the right atrium and the RV, is located inside the RV cavity and consists of leaflets, chordae tendineae, orifice (between the leaflets but also between the chordae interchordal spaces), annulus, and papillary muscles. The myocardium of the septum free wall is the platform of papillary muscles and should be considered an intrinsic part of the TV apparatus (*Figure* *3A* and *B*). The annulus is a tiny fibrous structure separating the right atrium from the RV and inserting leaflets and myocardium (*Figure 3C*). Unlike the mitral valve, the TV shows a septal leaflet that is attached to the ventricular septum by chordae tendineae or small papillary muscle. The three leaflets are separated by commissures, usually consisting of fan-like chordae. Although the TV is typically composed of three leaflets of unequal size, in many cases, two (bicuspid) or more than three leaflets may be present as anatomic variants in healthy subjects.^15^

The papillary muscles are usually three:

1. The septal Lancisi (conal) muscle, implanted in the ventricular septum. It gives origin to fan-like chordae that point to the antero-septal commissure, underneath which the membranous septum and the course of the His bundle are located.
2. The antero-papillary muscle, a big pillar in front of the antero-postero-inferior commissure, is attached to the septum through the moderate band;
3. The post-inferior papillar muscle consists of a group of pillars, which give origin to chordae tendineae joining the postero-septal commissure.

**Figure 4:**
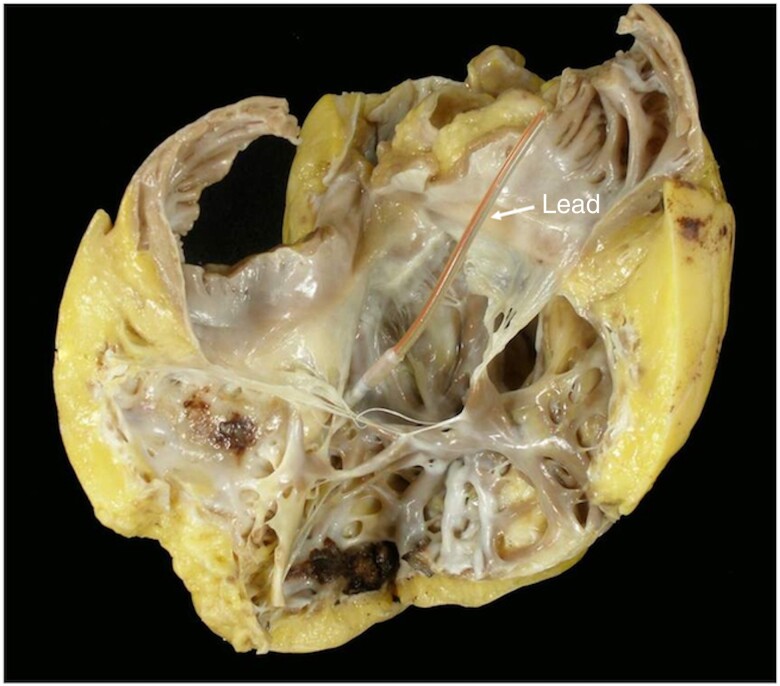
Post-mortem examination in a patient with a pacing lead showing a fibrotic process trapping the lead within the subvalvular apparatus of the tricuspid valve.

The largest muscle is typically the anterior papillary muscle with chordae supporting the anterior and posterior leaflets. The moderator band may join this papillary muscle. The posterior papillary muscle, which is often bifid or trifid, lends chordal support to the posterior and septal leaflets. The septal papillary muscle is variable: it may be small or multiple or even absent in up to 20% of normal patients. From an interventional perspective, the chordae may interact with catheters and devices, causing additional difficulties and challenges during transcatheter approaches for the TV.^15^ In addition, the mechanical properties and ultrastructure of normal human TV chordae tendineae consist of fairly straight collagen bundles that are made of networks of collagen fibrils and thus exhibit less extensibility than normal mitral valve chordae of comparable size.^15^ This may help explain the marked tethering that occurs with dilation of the RV or displacement of the papillary muscles. It has been shown on *post-mortem* examinations as well as *in vivo* 2D–3D echocardiographic studies in patients with device leads that a fibrotic process can entrap the device leads within the TV leaflet and/or subvalvular apparatus (*Figure 4*), resulting in various degrees of leaflet mal-alignment and mal-coaptation. Furthermore, these results explain why TLE can also cause damage to the TV apparatus and become a further mechanism of lead-mediated TV dysfunction.^16^

### Transvenous lead extraction worsening tricuspid regurgitation

The incidence of acute TRI following TLE is estimated to be in the range of 3.5–15%.^4–9^ The wide disparity may be a result of variability in the definition of acute TRI in each of these studies, differences in the baseline clinical characteristic of the study population, in dwell time of the extracted leads, and in tools used for the procedures. Recently, Park *et al*.^10^ reported that following TLE (mostly laser sheaths), significant TRI and TV damage were commonly detected (11.5%) by TEE particularly in patients with advanced lead age. More recently, Polewczyk *et al*^11^ found a 9.7% incidence of any progression in TR severity following mechanical TLE and severe TV damage in 2.5% of patients using routine both TTE and TEE examinations in all patients. Contrarily, most of the literature and expert consensus on TLE concentrate on injury to SVC, but not on TLE-related worsening of TR, reporting a much lower percentages of TV damage related to TLE and worsening TV function.^1–3^ It is likely that such large discrepancies in the assessment of the incidence of TRI following TLE results from the lack of routine echocardiographic evaluation before, after, and during the procedure. Moreover, while traumatic TR is often apparent immediately after extraction, the initial insult of TV with mild/trivial regurgitation immediately after TLE may lead to progressive severe valve dysfunction leading to development of severe TR requiring surgical intervention for symptoms of RV failure.^17^ Therefore, the present study, together with those mentioned above, is likely to provide a more realistic picture of the incidence of TV damage during TLE regardless of the tools used. Endocardial leads implanted on a long-term basis progressively develop fibrotic attachments to TV apparatus, as documented by autopsy studies and open heart surgical reports.^18^ Therefore, longer implant duration could lead to greater degree of fibrotic adhesion and an increased risk of TV injury during the extraction procedure. Not surprisingly, studies with shorter lead dwell times had a lower incidence of TR progression when compared with studies with longer dwell times. In the present study, implant duration was the only predictor of significant TRI. There were no other significant differences in baseline clinical and technical characteristics between patients with and without TRI. Our results are in line with the study by Park *et al*.^10^ in which only lead dwell time was an independent predictor of TLE-related acute TR.

The ELECTRa study showed that independent predictors of major complications, clinical failure, are related not only to the patient and lead profile but also to the use of powered sheaths, femoral approach, centre experience, and procedural volumes. Powered sheaths including laser and rotations mechanical sheath are controversially considered a risk factor for TLE-related acute TR aggravation. Franceschi *et al*.^5^ showed that the use of laser sheath as opposed to manual traction alone was the most powerful predictor of post-TLE significant TR. However, laser and rotational mechanical sheaths are usually reserved for difficult cases, possibly with a longer lead age and more extensive fibrotic adhesion between the lead and TV apparatus. To this regard, Polewczyk *et al.*^11^ found that the need for the use of second-line instruments (Evolution or TightRail, or loose catheter/loop catheter) was associated with significant worsening of TV function. Recent studies have shown that Evolution RL bidirectional rotational mechanical sheath (Cook Medical, Bloomington, IN, USA) is an effective and safe technique for the extraction of chronically implanted leads.^19–22^ However, there are limited data on the effect of bidirectional rotating mechanical lead extraction on the severity of TR with routine echocardiographic monitoring. The present study, for the first time, focused on the rate of worsening of TR following mechanical rotational TLE and their outcomes using echocardiographic evaluation including TTE pre–post-procedure and TEE monitoring during TLE in all cases.

We showed that in 94.3% of patients, transvenous rotational mechanical lead extraction had no negative influence on TV efficacy, and in 5.7% of patients, a significant TRI was noted, but a severe degree in 3%. Our results are in line with that observed in previous study using mostly laser technique.^5,10,11^ Therefore, the incidence of significant TRI and traumatic valve damage is not an uncommon complication after TLE, and it seems not correlated to the powered sheaths used (laser vs. rotational mechanical sheaths) when required because manual traction alone is ineffective. It is noteworthy to mention that the mean implant duration of our study was 93 ± 65 months, and 67% of the leads extracted had a passive fixation mechanism, which are significant risk factors for fibrous adherences, making TLE more challenging and potential associated to significant TRI. To this regard, Polewczyk *et al.*^11^ demonstrated that in addition to the need of multiple extraction tools and longer dwell times, a passive fixation mechanism is also a potential factor that predict a significant worsening of TV function.

According to our findings, patients who experienced significant TRI were more likely to have an infection indication. This association is difficult to explain as we did not observe an association between the presence of vegetations on TV or RV leads and significant TRI. In case of systemic infection, subclinical involvement of the tricuspid valve may occur resulting in susceptibility to damage following extraction. Furthermore, this association observed in the present study could also be due to a selection bias as infection is the most frequent indication.

An important issue is the further management of patients with significant TRI and TV damage after TLE, and the impact of severe TR on long-term prognosis.^4,11^ In the present study, two patients (1.2%) with severe TR after TLE developed refractory heart failure despite medical management during hospital stay and non-urgent TV replacement was performed in three patients (1.9%). The need for surgical intervention was rare and comparable to previous reports.^10,11^ According to our results, a severe increase of TR after TLE was an independent predictor of mortality along with severe systolic dysfunction and systemic infection.

Transcatheter approaches to treat clinically significant TR have emerged as alternative solutions to medical therapy and surgical interventions. The use of these treatment modalities in patients with existing CIED leads is not without risk, resulting in ‘jailing’ of leads outside the implanted valve stent, which may be impossible to extract in the future. In this subset of patients, there are two important considerations: does TLE damage the valve to a degree where a transcatheter TV intervention is no longer possible, and does TLE improve TR severity to the point where a TV intervention is no longer required? A ‘heart team’ approach that includes a lead management specialist should be strongly considered.^16^

### How to avoid worsening of tricuspid valve function?

An excessive pulling of the extracted lead during TLE to ensure a ‘rail effect’ for advancing the sheaths along the target lead could increase TV damage. It is our feeling and impression that excessive and uncontrolled pulling can cause extroflection and wrapping of the leaflet leading to an increase risk of TV damage. This dangerous phenomenon cannot be seen during fluoroscopy but is well visible by means of TEE monitoring. Controlled, non-excessive traction and gentle advancement of the mechanical sheath using the outer sheath over the target lead can reduce the risk of damage to the TV during the procedure. Simultaneous lead traction from above and below during TLE can also reduce excessive traction on the TV, reducing the risk of damage, especially in old leads.^23^ Co-operation with TEE monitoring may help warn the operator about potentially harmful situations leading to TV damage,^10,11^ outthought, but the extracted lead can be connected with chordae tendineae or even the head of the papillary muscle, and they can be damaged unnoticeably.^11^ For this reason, an echocardiographic re-evaluation should be mandatory not only during but also after the procedure.^17^

### Clinical implications

Physicians involved in TLE should give more attention to this possible complication as well as to the fear of damaging the SVC, especially in non-infected indications because still controversial. Cardiac evaluation including TTE pre–post-procedure and TEE monitoring during TLE could help in warning the operator about potentially harmful situations leading to TV damage. With respect to the prevention of TLE-related TV damage, the abandonment of unnecessary leads increases implantation duration and likely raises the risk of significant TRI, a consequence that should be avoided. Therefore, the alternative strategy of earlier TLE should be considered in expert centres.

### Study limitations

First of all, it is a retrospective single-centre study of patients undergoing mechanical TLE using exclusively the Evolution RL system with ancillary tools. No direct comparison was made between Evolution RL system and other techniques currently used for TLE. Hence, no conclusion can be drawn regarding the comparative safety of mechanical vs. laser sheaths. This is a single-centre study with a team experienced using bidirectional mechanical TLE sheaths; therefore, our results may not be widely applicable in less-experienced centres. The number of participants included in our study and the number of TRI events were relatively small, limiting the ability to detect further predictors of TRI besides dwell time of the leads. Pooling data from multiple centres who perform TEEs at the time of lead extraction would be valuable. However, this is the first report on the rate of significant changes in TR severity following mechanical rotational TLE and the outcome. Pacing electrodes may have shadowed the TV apparatus and caused underestimation of TR by TTE before lead removal. Possible imaging predictors for TRI by the pre-procedural TEE (e.g. the way the lead crossed the TV, whether it is tethered to the leaflets or the subvalvular apparatus) were not evaluated. Finally, the limited number of significant TRI is most probably crucial for statistical analysis. Our study protocol was designed to detect significant TRI occurring early after lead removal (before discharge) to allow for a close relationship between the procedure and the new TR. Therefore, although unlikely, TR that might have occurred later could not have been diagnosed. Larger series are needed for further analysis of the natural history and possible risk factors of the disease.

## Conclusions

Significant TRI assessed by echocardiographic evaluation was noted in 5.7% of patients and was linked to TV damage, indications of infection, and longer lead implant durations. The duration of lead implantation emerged as the sole predictor of significant TRI. Physicians engaged in TLE should exercise greater vigilance for this potential complication. Implementing strategies to mitigate the risk of TV damage is essential.

## Supplementary Material

euae191_Supplementary_Data

## Data Availability

The experimental data used to support the findings of this study are available from the corresponding author upon request.
