# Supplementary material for: Evaluation of tricuspid valve regurgitation following transvenous rotational mechanical lead extraction
Source: Europace. 2024 Jul 11;26(7):euae191. doi: 10.1093/europace/euae191 (PMC11282457; doi:10.1093/europace/euae191)
Supplement: euae191_Supplementary_Data [file euae191_supplementary_data.zip › Supplemental Table 1 .docx]

**Supplemental Table 1. Clinical, technical characteristics and outcomes of the patients with significant tricuspid valve regurgitation increase following transvenous lead extraction.**

| **Patient** | **Gender** | **Age** | **Underlying disease** | **Type of device** | **TLE Indication** | **Use of snare** | **Implant duration (months)** | **TR before TLE** | **TR following TLE** | **TV damage** | **TR Management** | **Indication to**  **Non-urgent TV replacement** | **Time from TLE and cardiac surgery (days)** |
| --- | --- | --- | --- | --- | --- | --- | --- | --- | --- | --- | --- | --- | --- |
| #1 | Male | 47 | DCM | ICD  single-chamber | Endocarditis | No | 143 | Trivial | Moderate | No | Medical therapy | - | - |
| #2 | Male | 85 | AVB | PM  single-chamber | Pocket infection | No | 25 | Mild | Moderate | No | Medical therapy | - | - |
| #3 | Male | 71 | Ischemic cardiomyopathy | ICD  single-chamber | Endocarditis | No | 86 | Mild | Severe | No | Medical therapy | - | - |
| #4 | Male | 77 | DCM | CRT-D | Endocarditis | No | 54 | Moderate | Severe | Flail  chordae | Medical therapy, not candidate for cardiac surgery due to significant comorbidities | TV damage, HF | - |
| #5 | Male | 75 | DCM | CRT-D | Endocarditis | No | 130 | Mild | Severe | Flail  chordae | Non-urgent TV replacement as planned procedure | TV damage,  HF | 8 |
| #6 | Male | 88 | AVB | PM  single-chamber | Systemic infection | No | 147 | Mild | Moderate | No | Medical therapy | - |  |
| #7 | Male | 74 | DCM | CRT-D | Systemic infection | No | 226 | Trivial | Severe | No | Non-urgent TV replacement as planned procedure | RV dilation,  asymptomatic | 63 |
| #8 | Female | 69 | AVB | PM  dual-chamber | Pocket infection | Yes | 259 | Mild | Severe | Leaflet avulsion | Non-urgent TV replacement as planned procedure | TV damage, asymptomatic | 21 |
| #9 | Male | 89 | AVB | PM  dual-chamber | Systemic infection | No | 148 | Mild | Moderate | No | Medical therapy | - | - |

AVB=atrio-ventricular block; DCM = dilated cardiomyopathy; HF=heart failure; ICD=implantable cardioverter defibrillator; CRT-D=cardiac resynchronization therapy-defibrillator; PM=pacemaker; TLE=transvenous lead extraction; TR=tricuspid regurgitation; TV=tricuspid valve
