# Supplementary figures and images for: Evaluation of tricuspid valve regurgitation following transvenous rotational mechanical lead extraction
Source: Europace. 2024 Jul 11;26(7):euae191. doi: 10.1093/europace/euae191 (PMC11282457; doi:10.1093/europace/euae191)

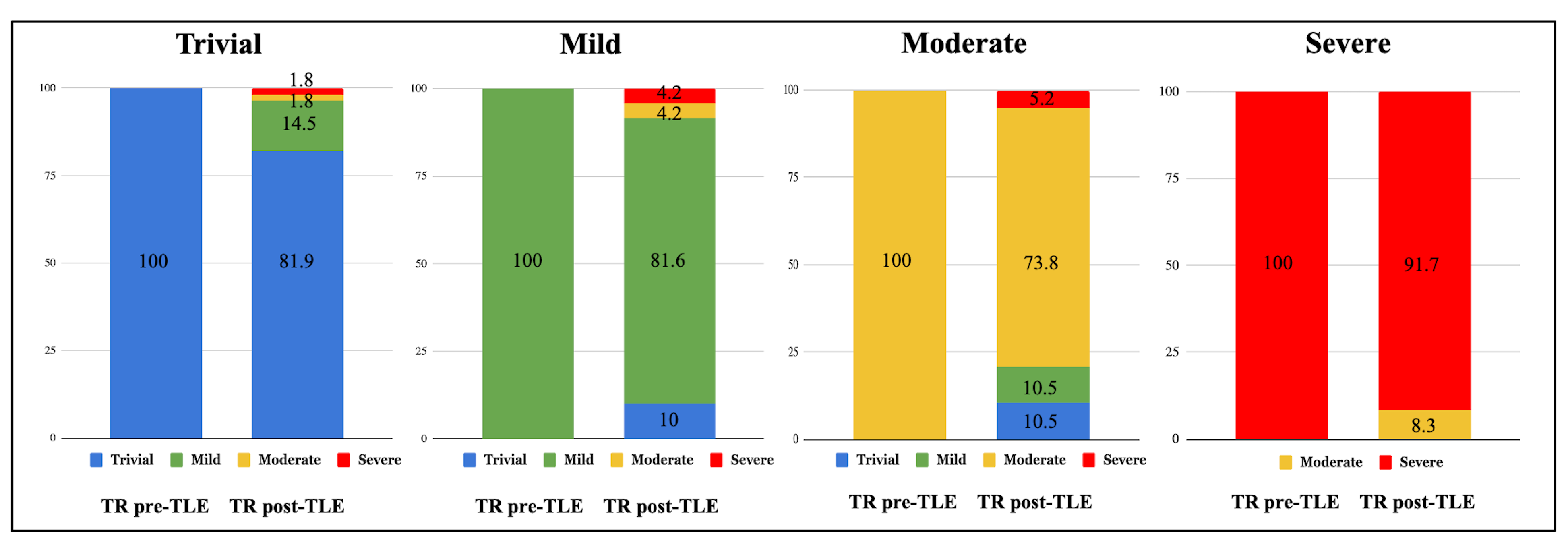

Supplement: euae191_Supplementary_Data [file euae191_supplementary_data.zip › Supplemental Figure 1.tiff]
